# The Effect of Price and Socio-Economic Level on the Consumption of Sugar-Sweetened Beverages (SSB): The Case of Ecuador

**DOI:** 10.1371/journal.pone.0152260

**Published:** 2016-03-30

**Authors:** Guillermo Paraje

**Affiliations:** Business School, Universidad Adolfo Ibáñez, Santiago de Chile, Chile; Universitat Jaume I, SPAIN

## Abstract

The objective of this article is to estimate the own-price, cross-price and income elasticities of demand for SSB in Ecuador, as an indispensable step for predicting a reduction in the consumption of said beverages caused by the potential implementation of taxes in Ecuador. In addition, the own-price, cross-price and income elasticities of sugar-free substitutes like mineral water and diet soft drinks and juices are also estimated. The data from the 2011–2012 ENIGHUR, which contains detailed information on household consumption and socioeconomic variables, was used. The estimates are done using Deaton’s Almost Ideal Demand System (AIDS) which accounts for differences in the quality of goods purchased. This demand system is estimated for different socio-economic groups, according to total household expenditure. The results reveal own-price elasticities for SSB between –1.17 and –1.33 depending on the socio-economic group, in line with the existing evidence for developed countries. Own-price elasticity for non-SSB is between -1 and -1.24. Income elasticities reveal that both SSB and non-SSB are normal goods with elasticities decreasing for higher socio-economic groups. These results show that the consumption of SSB is sensitive to price changes, meaning that the implementation of taxes on said beverages could be effective in reducing their consumption. The fact that non-SSB are also sensitive to price changes would indicate that subsidies could be implemented for the production of some of them.

## Introduction

The scientific evidence of the harmful effects that consuming sugar-sweetened beverages (SSB) has on health has accumulated over time and become increasingly convincing. A recent systematic review and meta-analysis examined the relationship between consumption of these beverages and diverse nutritional and health measures [1]. The review found that SSB consumption was associated with an increase in caloric intake (beyond the levels contributed by said beverages), an increase in body weight, an increase in prevalence of type 2 diabetes, hypocalcaemia, increased bone fractures, dental cavities, hypertension, etc. This type of correlations led some authors to think about the significant increase in future health system costs associated with overweightness/obesity, in developed as well as developing countries [2, 3]. A recent estimation of the burden of disease related to SSB concluded that worldwide 184,000 deaths/year are attributable to SSB consumption, 72% of them from diabetes [4]. Latin America and the Caribbean (LAC) is the region with the highest absolute mortality related to SSB consumption.

In the case of Ecuador, the burden of disease from SSB consumption is below LAC averages (2.4% of total deaths attributable to SSB consumption in Ecuador versus 4.2% in LAC). However, over the recent years the prevalence of overweightness and obesity has increased significantly, without distinction among sex, age or socioeconomic level, as shown by the recent National Ecuadorean Health and Nutrition Survey (ENSANUT) [5]. The excessive consumption of carbohydrates is 29% nationally, though there are differences among groups. For example, households from the poorest quintile show three times the excess consumption that households in the top quintile show. A significant part of this consumption is focused on SSB: average daily consumption of these beverages is 272 ml/day, though it reaches 359 ml/day for some groups, like men between the ages of 19 and 30. Per capita consumption of carbonated beverages (a significant proportion of SSB) increased 7% between 2009 and 2014 and it is expected to increase by an additional 6% through 2019, with consumption surpassing the levels of countries with incomes that are far higher than Ecuador’s such as Canada, the Netherlands, Switzerland, Austria and France [6].

Concerns about the health costs associated to consumption of SSB have led to an increased number of studies proposing economic measures–essentially taxes–to cut SSB consumption. While these measures are not the only ones proposed there is growing evidence of their potential effectiveness, as it is shown that SSB consumption is relatively sensitive to price movements. Their ultimate impact on health would depend, however, on the baseline tax level, obesity prevalence and SSB consumption [7]. A recent systematic review and meta-analysis of articles published between January 2000 and January 2013 on the effect of prices on the consumption of SSB revealed a joint own-price elasticity (that is, the percentage of demand reduction when faced with a 1% price increase) of -1.3 for SSB [7].

Estimates of own-price elasticities for developing countries are less frequent. In the aforementioned meta-analysis, only two from LAC are included. The first one is a study done for Mexico that estimated price elasticity for SSB and elasticity cross-referenced with milk [8]. Authors used three household surveys and a two-stage model (the first a probit model of the consumption decision and the second an Ordinary Least Squares–OLS–estimate to model intensity), controlling for socioeconomic variables. Significant negative price elasticity was found for SSB that grows over time (from -0.6 in 1989 to -1.1 in 2006). However, price data is not corrected for differences in quality, which may imply that average prices used by authors are endogenous.

The second study is for Brazil and the authors use the 2002–03 Household Spending Survey to estimate the own-price elasticity of SSB calories, using a log-log OLS model. After controlling for a series of socioeconomic variables, they find that the own-price elasticity is -0.85 [9]. The major limitation of this study is that, instead of using a two-stage model (as in [8]), it only considers price induced-changes in the purchase intensity of these beverages (conditioned to their purchase), meaning that total demand price elasticity and the potential dissuasive effect of a tax could be underestimated (apart from the mentioned endogeneity problem of not considering differences in quality of the purchased goods).

Recently, a study for Mexico [11] uses a lineal approximation of Deaton’s Almost Ideal Demand System (LA/AIDS), which considers differences in quality of purchased goods, to estimates own-price elasticities for SSB by socio-economic levels. The study finds that own-price elasticity is -1.16 for the general population with differences across income quintiles (higher quintiles tend to have more inelastic demands, though the relation is non-monotonic). No expenditure or income elasticity is reported.

There are no published studies estimating price or income elasticities for SSB in Ecuador. The objective of this article is to fill this gap and to add to the growing evidence in low and middle-income countries by estimating the own-price elasticity of demand for SSB as an indispensable step for predicting a reduction in the consumption of said beverages caused by the increase of taxes (currently at 10% of price including taxes). In addition, the own-price elasticity of sugar-free substitutes like mineral water, diet soft drinks and juices susceptible to potential subsidies, for example, is also estimated. Deaton’s Almost Ideal Demand System (AIDS) model, which controls for differences in quality of the purchased goods, is used to estimate these elasticities [10–12]. Moreover, cross-price elasticities (ie, the percentage of demand change in SSB when prices of non-SSB change by 1%) and income elasticities are also estimated. This is done for the entire population and for sub-groups of it, formed by stratifying household by total household expenditure, as a proxy of material affluence.

## Materials and Methods

### Data

The household-level consumption data used come from the 2011–2012 National Urban and Rural Household Income and Expenditures Survey (ENIGHUR), representative on the level of states, area (urban/rural) and on a local level for nine self-represented cities [13]. Said survey not only collects information on incomes and expenses, but also a broad-based socioeconomic description of households. In total, the 2011–2012 ENIGHUR contains information on 39,617 households, of which 1,383 were eliminated from the analysis (3.5% of original households) for failing to have any information on expenses.

Households are grouped into Primary Sampling Unit (PSU), statistical units defined from national census information and consisting of about 150 and 90 households in urban and rural households, respectively. The sample of ENIGHUR is drawn from a two-stage stratification process: the first stage is the selection of PSU, while the second stage is the selection of specific households within selected PSU. While the first-stage is non-random (the probability of PSU selection positively depends on the number of households within them), the second-stage is random [15]. Hence, households from the same PSU are geographically close.

Table 1 shows descriptive information for demographic variables that are relevant to the national total and for four specific geographic areas. It shows significant regional differences in terms of urbanization, with the Central and Southern regions in the country standing out for their low levels of urbanization. In addition, these are the regions where households have the smallest proportion of children under the age of 12 and heads of household with lowest educational attainments.

**Table 1:** Descriptive demographic variables.

*Variables*	*North*	*Coast*	*Centre*	*South*	*National*
*Number of households*	*11*,*67*,*687*	*16*,*47*,*110*	*4*,*31*,*765*	*4*,*24*,*042*	*36*,*70*,*603*
*Household average size*	*3*.*8*	*4*.*0*	*3*.*9*	*4*.*0*	*4*.*0*
*Proportion of urban households (%)*	*75*.*0*	*76*.*5*	*33*.*5*	*47*.*2*	*67*.*8*
*Average proportion of children less than 12 (%)*	*22*.*5*	*23*.*3*	*21*.*6*	*22*.*0*	*22*.*7*
*Average proportion of household heads with complete primary school or less (%)*	*47*.*5*	*50*.*1*	*64*.*9*	*61*.*3*	*52*.*2*
*Average proportion of household heads with complete secondary school or less (%)*	*33*.*5*	*34*.*0*	*23*.*1*	*23*.*5*	*31*.*4*
*Average proportion of household heads with some tertiary school -complete or not- (%)*	*19*.*0*	*15*.*9*	*12*.*1*	*15*.*2*	*16*.*4*

Source: own based on ENIGHUR 2011–2012

The “Classification of Individual Consumption According to Purpose” (COICOP) with seven digits (i.e. division, group and class of goods) was used for the SSB and non-SSB classification. In this context, SSB include tonic waters (COICOP 01.22.004), energy drinks (COICOP 01.22.005) and hydrating beverages (COICOP 01.22.006), colas and carbonated beverages (COICOP 01.22.007), refreshments (COICOP 01.22.009), other refreshments (COICOP 01.22.098), concentrated fruit juices (COICOP 01.22.101) and packaged natural fruit juices (COICOP 01.22.102). For their part, non-SSB are purified water with gas (COICOP 01.22.001), purified bottled water without gas (COICOP 01.22.002), purified water without gas in jugs (COICOP 01.22.003), light cola drinks or carbonated beverages (COICOP 01.22.008), other mineral waters (COICOP 01.22.097) and light natural fruit juices (COICOP 01.22.103).

Expenditures on these goods are registered for a week and then the amounts are estimated on a monthly basis so they can be compared with total household spending. All expenses are expressed in April 2011 dollars (no difference would be found if using another month as deflator base). In addition and for the purpose of the analysis, households are categorized as a function of their real total expenditures: the first “tercile” corresponds to 40 percent of households with the lowest real total monthly spending (quintiles 1 and 2); the second tercile corresponds to the next 40% of households (quintiles 3 and 4) and the last tercile corresponds to the 20% of households with the highest real total monthly spending (fifth quintile). This classification seems appropriate for middle-income countries like Ecuador, where the difference in the standard of living between the first two income quintiles does not appear to be significant. In fact, national poverty in 2011 surpassed the first quintile of the population (it was 28.7% and in rural areas 50.1%) [14]. Furthermore, some international agencies have begun to use this division to monitor their recommendations [15].

Once the real monthly spending on SSB and non-SSB is obtained and considering the amounts (in liters) that have been purchased, “unit values” for each one are obtained for households. These “unit values” are defined as average expenditure per unit acquired and they are frequently used as a proxy for the price paid [16]. The impossibility of identifying the specific good acquired (brand, product code, SKU, etc.) from the survey means having to add them in broader categories, such as those described above. While this addition is necessary to implement the survey (it would be unthinkable and impossible to collect exhaustive data on all of the goods acquired by a given household), it carries a cost in terms of the statistical analyses that are possible. For example, an increase in the unit value of a good would well entail higher prices paid for that good, or else a change in in the quality of the good acquired by a household [10]. Thus, the unit value masks a crucial decision within households: paying more for a given good (e.g. packaged fruit juice) or increasing the quality of the good (e.g. fruit juices in better containers).

Once these variables were defined and constructed, the existence of outliers in the unit values (e.g. unusually high or low values) was investigated. To this end, histogram and box plots were elaborated to observe this existence. Where the existence of said values was observed, it was decided that individual observations would be eliminated where one of the goods showed two standard deviations above or below the unit value, which entailed eliminating 961 households from the sample (around 2.5% of the sample with positive daily expenses) [17]. Notwithstanding the above, analyses were also undertaken considering the total sample, including those households with extraordinary unit values. In general, the estimates are relatively robust for these values. Table 2 shows the sampling averages for real total monthly expenses, those on SSB and non-SSB, the average amounts (liters) acquired and their unit values.

**Table 2:** Expenditure, consumption and unit values for SSB and non-SSB, by expenditure terciles.

*Variables*	*Tercile 1*	*Tercile 2*	*Tercile 3*	*National*
*Average household total expenditure (in April 2011 US dollars)*	348.6	754.0	1,842.2	780.1
*Average proportion of households with SSB expenditures* (*)	31.8	48.2	48.0	40.0
*Average household expenditure on SSB (in April 2011 US dollars)* (*)	6.1	7.7	9.4	7.5
*Average quantity (in liters) of SSB* (*)	8.9	11.1	12.5	10.7
*Average unit value per liter of SSB (in April 2011 US dolars)* (*)	0.79	0.80	0.81	0.80
*Average proportion of households with non-SSB expenditures* (*)	8.6	16.4	23.1	13.9
*Average household expenditure on non-SSB (in April 2011 US dollars)* (*)	4.9	6.1	7.7	6.3
*Average quantity (in liters) of non-SSB* (*)	59.8	68.2	72.3	67.3
*Average unit value per liter of non-SSB (in April 2011 US dolars)* (*)	0.30	0.30	0.33	0.30

(*) *Estimated on household with positive consumption of relevant goods*

Source: own based on ENIGHUR 2011–2012

As can be observed, there are significant differences in the level of average total real spending among terciles. Households from higher socioeconomic sectors consume both SSB as well as non-SSB with greater frequency. They consume both beverages more on average and pay a higher unit value (presumably because they buy higher quality beverages).

### Method

The estimation of own-price elasticities of SSB and non-SSB, in addition to the respective income elasticities (i.e. the percentage increase in demand in the context of a percentage increase in total household income, proxied by household budget) and cross-price elasticities (i.e. percentage increase in the demand for a given good when the price of another is increased) of the goods is conducted using Deaton’s AIDS, which considers differences in the quality of the goods purchased by households.

As mentioned above, it is virtually impossible to work with individual prices based on the data contained in household expenditures surveys, which is why “unit values” are used. As noted, the choice of items of differing qualities within the same good affects the unit value. In this context, this value is chosen by consumers (upon deciding on the quality of the good), contrary to what happens with the price, which they have no influence over at all.

Deaton proposes a system of equations that, based on certain premises, allows one to estimate demand elasticities while controlling for differences in the quality of goods [10–12]. One of the initial premises is that there are no price variations within a geographic area near the households. Thus, the variations observed in the unit values for the households in a given area are due to differences in the quality of the goods acquired. This premise, crucial for the analysis, requires that the households considered as part of a same area be physically close to each other and for them to have reported their expenditures in similar periods of time (especially in inflationary contexts).

Concretely, Deaton proposes estimating two equations:

$$w_{ghc}=\alpha_{g}^{0}+\beta_{g}^{0}lnx_{hc}+\gamma_{g}^{0}z_{hc}+\sum_{J=1}^{N}\theta_{gJ}\;{lnp}_{Jc}+(f_{gc}+u_{ghc}^{0})$$ (1)

$$ln{uv}_{ghc}=\alpha_{g}^{1}+\beta_{g}^{1}lnx_{hc}+\gamma_{g}^{1}z_{hc}+\sum_{J\;=1}^{N}\psi_{gJ}\;lnp_{Jc}+u_{ghc}^{1}$$ (2)

where *w*_*ghc*_ is the proportion of total expenditure (budget share) for household *h*, located in area *c*, allocated to acquiring good *g*; *x*_*hc*_ is total household budget; *p*_*Jc*_ is the price of the N goods that the household can buy and which it is supposed does not vary within area *c*; *z*_*hc*_ is a vector of household characteristics and *f* is an error associated with the area (cluster), while *u* is an idiosyncratic error (i.e. for each home). Thus, the proportion that each household spends on each good depends on the household’s total budget, the inherent characteristics of the household and the price of all goods that said household deals with. In Eq (2), *uv*_*ghc*_ is the unit value of the good *g* for household *h* (in area *c*) and it is a function of the same variables that affect *w*_*ghc*_ (except for the cluster level error). As Deaton explicitly states, Eqs (1) and (2) arise from “a model of consumer behavior in which households choose how much of a commodity to buy and in what quality or grade. Commodities are considered as collections of heterogeneous goods within which consumers can choose more or less expensive items, so that the unit value of a commodity, the price paid per physical unit, is a matter of choice. Both quantity and quality choice are functions of household income, household characteristics, and price” [14]. Spatial variations in unit values among different clusters provides an identification strategy, avoiding endogeneity of prices by considering differences in quality choice. This is the main advantage of Deaton’s AIDS over other alternatives of estimation.

As the prices of goods are not observed these equations cannot be estimated directly. Nevertheless, the premise that households in a given area are faced with the same prices allows these equations to be estimated in three steps [17]. The first consists in controlling for the socioeconomic characteristics of households in budget shares and unit values. The second consists in using the adjusted budget shared (from the previous stage) and unit values, averaged by area, to estimate errors with measurement errors (error-in-variable models) between areas (clusters). Lastly, the third stage separates the effect of quality and price. Thus, these three stages allow one to estimate total corrected price elasticities and cross elasticities for quality differences [12, 18].

Price elasticities are:

$$\varepsilon_{gj}=\theta_{gj}/w_{g}-\delta_{gj}$$

where *δ*_*gj*_ is the Kronecker delta (equal to 1 if g = j or to 0 otherwise) and budget shares are assessed at their means. The coefficient $\beta_{g}^{0}$ represent the elasticity of quantity demanded with respect to total expenditure [19].

The unit values of both SSB and non-SSB assigned to each household correspond to the average of the unit values in the given geographical area that households belong to. The geographical area must be small enough to consider households that are faced with the same prices of the goods under consideration (it is considered that households in the same area have the same sales points available to them and that they face the same purchase conditions). The smallest, most homogeneous geographical areas in the ENIGHUR are PSU and so, they are chosen.

The *z*_*hc*_ vector of household characteristics is composed by the natural logarithm of household size (the hypothesis is that households with a larger number of people tend to consume more goods, regardless of the amount of them consumed); gender of the head of household; three dichotomous variables that indicate the level of education attained by the head of household (heads of household with up to primary school completed; those with up to secondary school completed, and those with a certain amount of higher education–complete or incomplete); and dichotomous variable for the area (rural/urban). In addition, three dichotomous variables for the country’s regions (North is the region by omission); three dichotomous for the quarter of the year in which the household reports its spending (this is to control for the seasonal nature of expenditures and the first quarter is the reference quarter) are also considered.

The demand system is estimated using STATA 14 considering sampling design and sampling weights.

## Results

**Fig 1:**
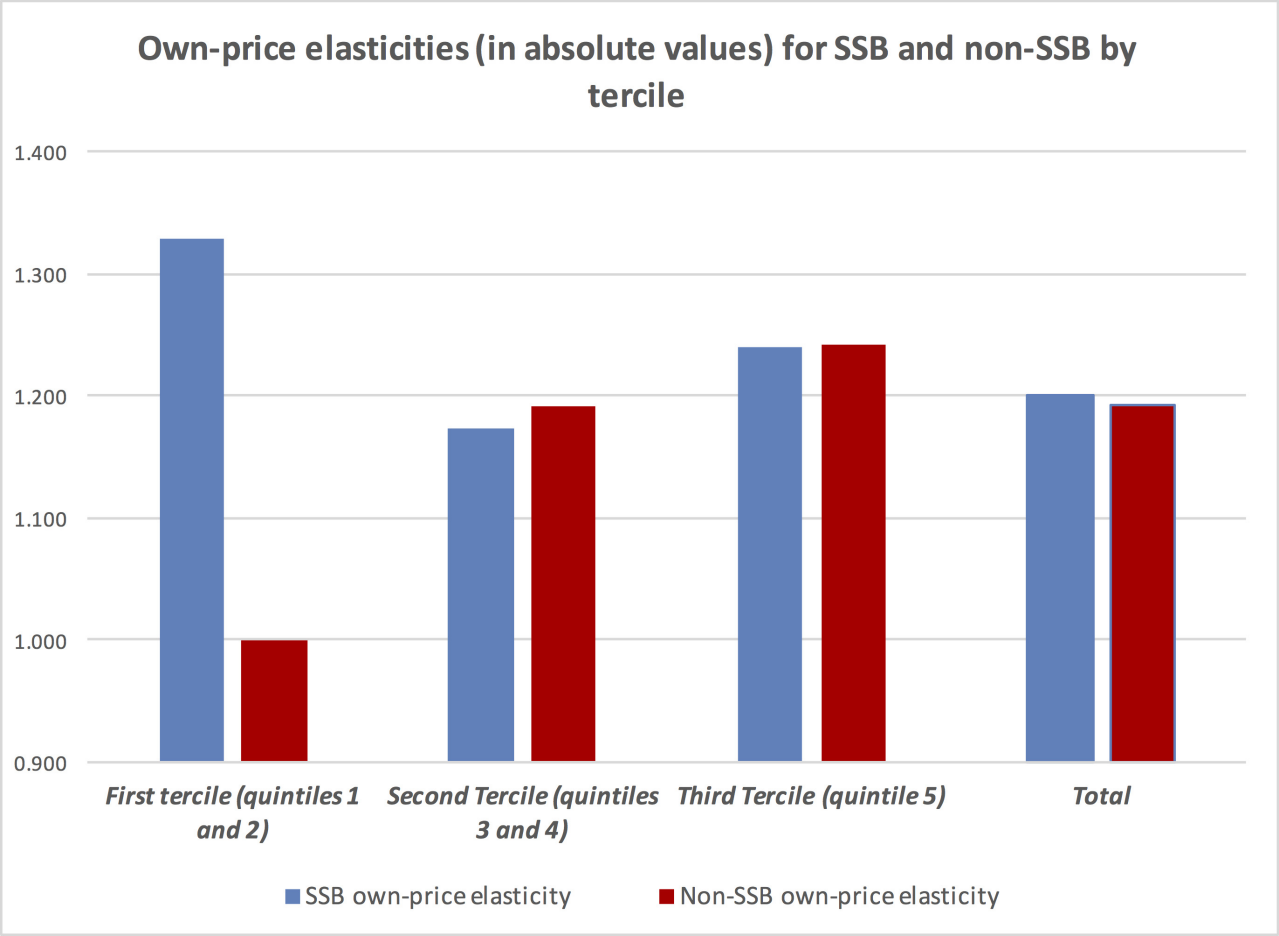
Own-price elasticities for SSB and non-SSB by budget tercile.

**Fig 2:**
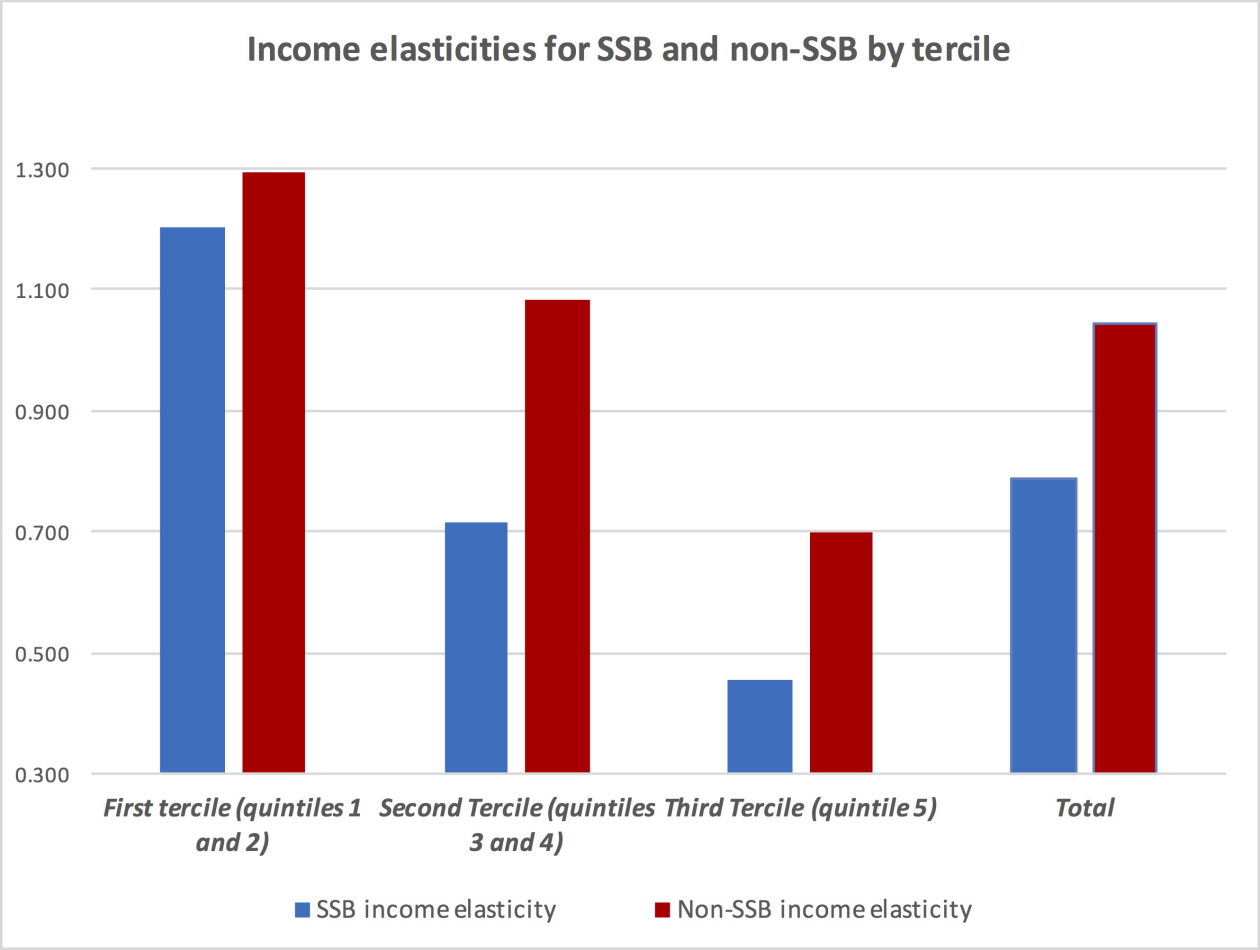
Income elasticities for SSB and non-SSB by budget tercile.

Table 3 presents the own-price elasticities, income and cross-price elasticities for SSB as well as non-SSB using Deaton’s AIDS, along with their standard errors obtained from carrying out a bootstrap test on the sample 150 times (considering sampling stratification). Results are presented by tercile and for the aggregate national level. **Fig 1**shows own-price elasticities for terciles and also national level, while Fig 2 presents income elasticities also for terciles and national level.

**Table 3:** Own-price, cross-price and income elasticities for SSB and non-SSB.

***First Tercile (quintiles 1 and 2)***			
***SSB Elasticities***	**Elasticity**		**SE** **(1)**
Own-price elasticity	-1.328	(***)	0.099
Income-elasticity	1.202	(***)	0.063
Cross-price elasticity	0.614	(***)	0.223
***Non-SSB Elasticities***			
Own-price elasticity	-1.000	(***)	0.087
Income-elasticity	1.295	(***)	0.103
Cross-price elasticity	-0.053		0.033
***Second Tercile (quintiles 3 and 4)***			
***SSB Elasticities***	**Elasticity**		**SE** **(1)**
Own-price elasticity	-1.173	(***)	0.062
Income-elasticity	0.715	(***)	0.070
Cross-price elasticity	0.484	(***)	0.125
***Non-SSB Elasticities***			
Own-price elasticity	-1.192	(***)	0.038
Income-elasticity	1.081	(***)	0.144
Cross-price elasticity	0.011		0.021
***Third Tercile (quintile 5)***			
***SSB Elasticities***	**Elasticity**		**SE** **(1)**
Own-price elasticity	-1.240	(***)	0.090
Income-elasticity	0.456	(***)	0.051
Cross-price elasticity	0.483	(***)	0.139
***Non-SSB Elasticities***			
Own-price elasticity	-1.242	(***)	0.045
Income-elasticity	0.698	(***)	0.071
Cross-price elasticity	0.049		0.033
***Total***			
***SSB Elasticities***	**Elasticity**		
Own-price elasticity	-1.201	(***)	0.056
Income-elasticity	0.788	(***)	0.021
Cross-price elasticity	0.550	(***)	0.116
***Non-SSB Elasticities***			
Own-price elasticity	-1.192	(***)	0.028
Income-elasticity	1.043	(***)	0.031
Cross-price elasticity	-0.005		0.018

(***) Significant at 1%

(**) significant at 5%

(*) significant at 10%

(1) Bootstrap standard errors after 150 replications

Own-price elasticities by terciles are negative and statistically significant in all cases, as they are for the national aggregate level. In the case of SSB the national elasticity is -1.2, while it is -1.19 for non-SSB. In the first case, the highest own-price elasticity (in absolute values) is found for the first tercile (quintiles 1 and 2), equal to -1.33; while the lowest is for the second tercile (quintiles 2 and 4), equal to -1.17. For non-SSB, the lowest own-price elasticity (in absolute values) is for the first tercile, equal to -1; while the highest it is found for the third tercile (-1.24).

Both goods are normal (i.e. income elasticities are positive), though the non-SSB have a significant higher elasticity than that of SSB. At the national level, the first equals 1.04 (i.e. a 10% increase in real household income increases demand for non-SSB by 10.4%), while the second equals 0.79 (i.e. a 10% increase in real income increases demand for SSB by 7.9%). The income elasticities by terciles show declining, statistically significant elasticities in both cases, with significant differences between tercile 1 and the others. This implies that a proportional increase in household income would increase consumption of SSB and non-SSB proportionally for households belonging to lower socioeconomic strata (i.e. lower spending terciles). Income elasticities over 1 represent goods considered as “luxury or superior”, while goods with income elasticities under 1 represent goods perceived as “necessary”. In the case of both goods, decreasing income elasticities over terciles imply that both goods are perceived as superior by relatively poorer households, reflecting a higher willingness to increase expenditures in these goods if household incomes go up or to decrease expenditures otherwise.

Third, cross-price elasticities are significant and positive only for SSB, showing both goods are substitutes. At the national level it is equal to 0.55 (i.e. a 10% increase if the relative price of non-SSB, increases SSB consumption by 5.5%). The substitution relationship is higher for lower socio-economic groups. No such a relationship is found for non-SSB (i.e. relative price increases/decreases in SSB would not affect non-SSB consumption).

## Discussion

Currently, Ecuador has a tax on SSB that is at 10% of price (including taxes) and could raise an estimate of USD 58 million (according to estimates from Ecuador’s Ministry of Public Health). Such a tax, implemented in 2001 has been unable to stop the increasing consumption of SSB, which has been growing at 3% annually between 2009–2014 [19]. There are neither estimates about the potential impact that an increase in the tax on SSB would have on consumption and on fiscal revenues, nor of the impact that subsidies to SSB substitutes could have. This study provides elasticity estimates as a first, necessary stage for such an assessment.

The results are in line with the results of other international studies [8–11, 26], but using a solid and robust methodology that controls for differences in quality of the goods purchased, and considering expenditures for different socio-economic groups to account for distinct purchase patterns. The own-price elasticities obtained show that an increase in the price of SSB (induced by a tax change) could significantly reduce their consumption. By controlling for quality choice, these elasticities consider substitution within the SSB category and provide more accurate estimates of demand response to price changes.

In the case of income elasticities, they show both goods are normal though non-SSB have a much higher elasticity than SSB. In this case, lower socio-economic groups have larger elasticities than higher socio-economic groups revealing that they are more prone to increase (decrease) consumption of goods with increases (decreases) in household incomes. This is the same as saying that the Engel curves for SSB are upward sloping but with a negative second derivative [22].

Given the inexistence of an epidemiological model for Ecuador similar to the one considered in [20], it is difficult to know what the precise impact will be on overweightness, obesity and conditions associated with these, though there is solid evidence for other countries that these taxes have a positive impact on health [21]. However, it is possible to approximate certain effects on consumption based on the results obtained and reasonable assumptions.

The per capita consumption of off-trade carbonates in 2014 in Ecuador was 51 liters [19]. The consumption of other SSB like RTD teas, juices and concentrates, and energy drinks, which was another 16 liters per person, approximately, would have to be added to that. Given these numbers, a 10% tax increase on SSB could reduce demand by about 12% (considering the price elasticity corrected for the quality differences obtained in this study). This means that consumption of SSB would go from the aforementioned 67 liters per capita per year to less than 59 liters.

This supposes that the tax is passed on to prices in full (pass-through equal to one), something that depends on the companies. Though it is not possible to know this, there are recent cases that would appear to indicate that the pass-through could even be more than one (ie, companies raise prices even more than the tax). The experience in Mexico after taxes on SSB were imposed in early 2014 was that the price of SSB went up even more than was to be expected from the tax [22]. This, which a priori would appear counterintuitive, is consistent with the optimal economic behavior of companies operating in highly concentrated markets [23]. For the case of carbonated beverages in Ecuador, the Herfindahl-Hirschman Index (HHI), a measurement commonly used to gauge market concentration, is equal to 0.39, considerably higher than the high concentration threshold defined by the US Justice Department when it analyzes mergers [24]. HHIs are similar or higher in the case of other types of SSB. Thus, evidence would indicate that an increase in tax for SSB of 10%, for example, could increase the price of these goods by even more than 10% and, given their elasticity, reduce their consumption substantially more than 12%, an estimate that would become a lower-bound threshold here.

The fact that households in the lowest socioeconomic levels have price elasticities that are higher than those of the upper levels indicates that the greater reduction in the consumption of SSB would be among this group of households. Contrary to what happens in the taxation of other harmful substances like tobacco, which have a heavily addictive component, the tax on SSB allows households to easily “escape” from the tax by consuming alternatives not covered by the tax. In such a case, taxes on SSB will not be regressive as they usually are in the case of tobacco [25]. One could even consider subsidies for healthy alternatives to increase their consumption and to compensate relatively poor households for the higher tax burden on SSB. It is possible that the effect on the economic welfare of these households would be positive in the face of this mix of fiscal instruments and at any rate that they will have a positive and greater impact on their health [21, 26].

The income elasticities obtained suggest that changes in households’ income levels would increase demand for SSB, especially among households in lower socioeconomic levels, which have higher elasticities. This means that the taxes, designed to lower consumption of SSB, should be designed in such a way that an increase in income thanks to economic growth does not increases the affordability of these goods with the passage of time. In this context, if the tax is specific (a monetary amount per liter) it would be desirable to have an automatic adjustment mechanism that could update the tax amount in line with inflation and increases in incomes. On the other hand, with an ad-valorem tax (as the current existing tax in Ecuador) a periodical review of the tax rate would be needed to adjust for income changes.

The study presented contains certain limitations that must be considered. The main one is that it does not consider consumption of all potentially caloric beverages. First, only the off-trade consumption of SSB (and non-SSB) is considered, that is, without considering restaurants, pubs and bars. While off-trade consumption represents the largest portion of the volume of SSB consumed (for carbonates, it represents 80% of total consumption), it represents slightly less than half of what households spend on this type of beverages [19]. It is difficult (if not impossible based on expenditure surveys like the one presented here) to calculate the value of the good acquired and the value of the services associated with on-trade consumption. While it is inappropriate to suppose that price elasticity of on-trade consumption is equal to that of off-trade consumption, one can say that a tax increase would also have an impact on on-trade consumption and that the aggregate drop in consumption of SSB would be greater than estimated here.

Second, possible SSB substitutes are not considered. For example, the consumption of coffee, tea, milks, yoghurts, etc. are not taken into account (unlike, for instance, [9, 10]). The reason for this is that the ENIGHUR data does not allow one to distinguish whether these goods have added sugar nor not. While it is true that sugared yoghurts and milks, for examples, have beneficial nutrients that SSB do not possess, it is also true that some of them contain amounts of sugar that make their consumption inappropriate for some people. The same can be said of tea and coffee. Given the impossibility of separating these goods according to whether they have added sugar or not, it was decided that they be left out. Other potential substitutes, such as beer are not considered as they have different regulations (e.g. license to sell alcohol, age-limits to buy it, etc.) and taxed separately.

Lastly, it does not consider tap water among the non-SSB. In countries like Ecuador, with relatively high coverage of improved water supplies (94% nationwide, according to [27]), it is reasonable to assume that an increase in the relative price of SSB would increase the consumption of drinking water from the tap. There is no information from ENIGHUR about the total household consumption of tap water for drinking, neither of its price.

Despite these limitations, the evidence presented here suggests that an increase in tax on SSB could significantly reduce their consumption in Ecuador and act as an important public health tool. The relatively high burden of disease related to SSB consumption (2.4% of all adults’ deaths in 2010) can be reduced with this economic tool that it is regularly used to limit the consumption of other harmful goods (e.g., tobacco, alcohol, diesel, etc.). The collection of said tax could be used to finance complementary tools not related to prices, such as education programs in schools and among at-risk groups.
